# Do Experiences Studying Abroad Promote Dialectical Thinking? Empirical Evidence From Chinese International Students

**DOI:** 10.3389/fpsyg.2021.595935

**Published:** 2021-05-28

**Authors:** Xiaomeng Hu, Yang Wang, Shanhui Liao, Kaiping Peng

**Affiliations:** ^1^Department of Psychology, Renmin University of China, Beijing, China; ^2^Department of Psychology, Tsinghua University, Beijing, China

**Keywords:** multicultural experiences, dialectical thinking, human universals, cultural differences, Chinese international students

## Abstract

Our current work seeks to provide direct empirical evidence on whether Chinese international students’ experiences studying abroad promote dialectical thinking. We collected behavioral data from 258 Chinese international students studying in multiple regions. We found that there was a main effect among the four conditions (i.e., studying abroad, exposure to foreign culture, hometown, and typical day). More specifically, when primed with studying abroad or typical day (relative to hometown culture), participants were more likely to show tolerance for contradiction by deeming both sides of contradictory scientific statements as convincing and rating them more favorably. Therefore, it is plausible that Chinese international students’ experiences studying abroad promote their dialectical thinking. More work is needed to further this line of research by (1) extending these effects with other measures of dialectical thinking such as perception of interconnectedness and prediction of change, (2) adopting differing paradigms to provide more robust findings, and (3) probing the underlying processes as to why experiences studying abroad promote dialectical thinking.

## Introduction

International education has been flourishing for several decades as the process of globalization has broadened and deepened. According to Open Doors Data from the Institute of International Education, China has become the leading source of international students in Western countries (IIE, 2020). For example, international students from China account for 42% of all international students in United States universities and colleges (IIE, 2020). Psychologists are increasingly interested in examining the psychological impacts of Chinese International students’ experiences studying abroad. Existing work has mainly touched upon the following domains. First, Chinese international students face great challenges in studying in host countries, a large body of work has looked at their mental health and emotional well-being (Liu, 2009; Mori, 2011; Choy and Alon, 2018; Zhuang et al., 2019), such as academic stress (Wei et al., 2007; Li et al., 2010), loneliness (Sawir et al., 2007), stress and coping (Xu et al., 2019), depression and anxiety (Sümer et al., 2014), and life satisfaction (Sam, 2001). Second, most Chinese international students are sojourners who temporarily reside in a foreign country mainly for education purposes, thus inevitably facing cultural adaptation issues. Extant work has studied their acculturation experiences (Kashima and Loh, 2006; Spencer-Oatey and Xiong, 2006; Wang and Mallinckrodt, 2006; Smith and Khawaja, 2011; Yan and Berliner, 2011), cultural identity (Maeder-Qian, 2018), social connectedness (Hendrickson et al., 2011; Cao et al., 2018), social interactions (Trice, 2004; Billedo et al., 2020), and sense of belonging, etc. Third, as a minority and marginalized social group, Chinese international students also face the detrimental consequences of stereotypes and prejudices (Poyrazli and Lopez, 2007; Ruble and Zhang, 2013). Fourth, prior work also argues that experiences studying abroad should exert profound psychological impacts on International students’ academic life, social life, and their personality development (Zimmermann and Neyer, 2013; Gieser, 2015). For instance, evidence suggests studying abroad experiences exert positive influences on extraversion, openness, and agreeableness, and negative influence on neuroticism (Zimmermann and Neyer, 2013). Moreover, studying abroad programs enhance college students’ foreign language learning and cultural adaptation self-efficacy (Milstein, 2005; Cubillos and Ilvento, 2012). Additional aspects of investigation involved their learning styles (Wong, 2004), migration intentions (Hazen and Alberts, 2006), multicultural personality (Oudenhoven and Zee, 2002), and decision making process (María Cubillo et al., 2006).

### The Psychological Outcomes of Multicultural Experiences

Increasing number of individuals hold multicultural experiences as they engage in differing cultural contexts or interact with people from differing cultural backgrounds. Cultural psychologists contend that the impacts of individuals’ multicultural experiences on their mental processes and behavioral patterns are mixed, paradoxical, and even seemingly contradictory (Hu et al., 2021). Past work suggests that the breadth and depth of multicultural experiences promote creativity (Leung et al., 2008), cognitive flexibility (Ritter et al., 2012), large-scale cooperation (Buchan et al., 2009), generalized trust (Cao et al., 2014), etc. However, multicultural experiences may also lead to greater outgroup prejudice (Sparkman et al., 2016), unethical behavior (Lu et al., 2017) and even exacerbate clashes of civilizations (Huntington, 1997). A recent review article provides researchers and practitioners a systematic review and a novel theoretical framework to tap into the psychological impacts of multicultural experiences as well as the underlying mediators and potential moderators of those effects (Maddux et al., 2020). We argue that Chinese international students’ studying abroad experiences can be deemed as one form of multicultural experiences thus the abovementioned effects would also be applicable to the cultural group of Chinese international students.

### The Construct of Dialectical Thinking

Since the two renowned cultural] psychologists Peng and Nisbett (1999) put forward the concept of dialectical thinking in a highly influential article published in the American Psychologist two decades ago, a large body of work that has been done to further this line of research (Ma-Kellams et al., 2011; Wang et al., 2016; Spencer-Rodgers et al., 2018). Nisbett et al. (2001) contend that dialectical thinking contained in Eastern culture is not equivalent to the dialectic of Hegel and Marx in Western philosophy. Dialectical thinking is a cognitive style or cognitive frame, which contains a series of closely linked cognitive processes (Spencer-Rodgers et al., 2018). Dialectical thinking represents the ideological tradition based on Eastern philosophy, especially the lay epistemology of Taoism (Spencer-Rodgers et al., 2018). Dialectical thinking is not static, but domain-specific and context-dependent. It is influenced and shaped by a variety of factors, including sample characteristics (age, gender, development stage, and cultural adaptation, etc.), specific domains (self, cognition, emotions, decision-making, and intimacy, etc.) and contextual factors (Spencer-Rodgers et al., 2018).

Dialectical thinking consists of three core principles: principle of change (i.e., reality is a dynamic process), principle of contradiction (i.e., contradictory things can exist simultaneously), and principle of holism (i.e., everything is interrelated) (Spencer-Rodgers et al., 2018). First, the expectation of change means that East Asians believe that all phenomena and events in the world change in a cyclical manner, so the expectation of change is inevitable (Spencer-Rodgers et al., 2018). However, Westerners’ expectations of change tend to be stable or linear, either gradually increasing or decreasing (Spencer-Rodgers et al., 2018). Second, tolerance for contradictions means that East Asians consider contradiction to be a natural, intrinsic, and unavoidable feature of all beings. Therefore, East Asians can accept phenomena that seem to be contradictory, and do not need to resolve them. East Asians prefer moderation or compromise, which is similar to the golden thought advocated by Confucian culture (Spencer-Rodgers et al., 2018). However, Westerners reject seemingly contradictory phenomenon, believing that contradiction violates the formal logic proposed by Aristotle (Nisbett et al., 2001), so contradiction must be resolved or integrated. Third, the perception of interconnectedness means that East Asians pay more attention to the relationship between the whole and the parts of things. East Asians believe that all objects, people, systems, and ideas have a permanent relationship (Spencer-Rodgers et al., 2018). However, Westerners pay more attention to the focal object and ignore the specific context of the object (Nisbett et al., 2001).

### Research Gap

There is some research gap in the current literature. First, despite the huge numbers and rapid rate of increase of Chinese international students across the globe, empirical research on the psychological antecedents and consequences of studying abroad, especially theoretical formulation and empirical work on how and why studying abroad experiences might reshape their psychological processes and behavioral patterns has been limited. More work is clearly needed to better our understanding on International students’ multicultural experiences would alter their way of thinking, feeling, and behaving. Second, previous work has examined the psychological impacts of individuals’ multicultural experiences on their creativity, generalized trust, personality development, self-efficacy, and outgroup prejudice, etc. Extant work, however, has not looked at how Chinese international students’ multicultural experiences affect their thinking styles in general and dialectical thinking in particular. Third, although a large amount of work has looked at between-culture variations and within-culture variations on dialectical thinking, no prior work has examined how and why individuals’ multicultural experiences would shift around dialectical thinking in a dynamic manner, taken together, despite the growing body of work pertaining to the psychological outcomes of multicultural experiences, mental health and psychological well-being of Chinese international students and the large body of work on dialectical thinking, there has been little work directly looking at how and why Chinese international students’ multicultural experiences affect their systems of thought and dialectical thinking. Our current work therefore sought to reduce this research gap by directly examining how Chinese international students’ experiences studying abroad in diverse regions would influence their system of thought.

### The Current Research

Past research has well-documented cultural differences in tolerance for contradiction. Peng and Nisbett observed that members of East Asian (i.e., Chinese, Japanese, and Korean) are more likely to display tolerance and acceptance of conflicting elements or events. As they have articulated in their article, they asserted that “holistic thinkers tend to engage in reasoning involving contradictions that tolerate opposites, whereas analytic thinkers tend to engage in reasoning involving contradiction that chooses one of two opposing propositions (Peng and Nisbett, 1999; Spencer-Rodgers et al., 2018). Little work, however, has looked at whether this pattern can be shifted by increasing multicultural experiences or by temporarily inducing corresponding cultural experiences in host country among frequent cultural movers (e.g., International students). Drawing upon the dynamic constructivist approach (Hong et al., 2000), it would be interesting to see if multicultural experiences and dialectical thinking dynamically interplay with each other. For instance, would individuals with richer multicultural experiences (e.g., breadth and depth) tend to be more dialectical or less dialectical? Would studying abroad experiences enhance or reduce dialectical thinking? Would priming studying abroad experiences enhance participants’ tolerance for contradiction? There are three dimensions within dialectical thinking. Considering that measuring all three tasks altogether would produce confounding effects such as our manipulation is not powerful enough to affect all three tasks or if we do detect the hypothesized effect we cannot make exact casual claims. We therefore only focus on tolerance for contradiction for the current investigation.

We propose two plausible research hypotheses. Considering that the cultural mobility patterns of Chinese international students are from China to mostly Western countries such as United States, United Kingdom, Canada, Germany, and Australia, etc., it is plausible that Chinese international students would adopt those cultures’ typical way of thinking, which is analytical thinking, as the acculturation process broadens and deepens. Conversely, it is also likely that studying abroad experiences make Chinese international students endorse multiple cultural perspectives which are to some extent incompatible and contradictory. Therefore, they have to adopt dialectical thinking to meet the needs of the demanding acculturation process. We therefore make the following two competing predictions:

H1: Priming studying abroad experiences encourage Chinese international students to show greater tolerance for contradiction, relative to priming exposure to foreign culture, hometown culture, and control condition.
H2: Priming studying abroad experiences encourage Chinese international students to show less tolerance for contradiction, relative to priming exposure to foreign culture, hometown culture, and control condition.

Based upon extant work and our theoretical formulation, we argue that the second hypothesis is more theoretically plausible. Existing work has found that multicultural experiences can enhance cognitive complexity, increase openness personality, and reduce outgroup bias. One of the core features of dialectical thinking is tolerating contradictions, which represents that two things or two viewpoints that seem contradictory can co-exist and be compatible with each other. Therefore, we infer that when individuals encounter or interact with foreign cultural members, their cognitive flexibility and openness to experiences will likely increase. Thus, their degrees of tolerance for contradictions may be enhanced through increased integrative complexity (Tadmor et al., 2012). Alternatively, cultural identity threats may arise due to foreign cultural inflow induced by recalling studying abroad experiences (Morris et al., 2011), therefore participants’ Chinese way of thinking may be enhanced to meet their cultural identity needs. Our current work will provide direct evidence to address this empirical question.

## Materials and Methods

### Participants and Procedures

Through an online platform we recruited 265 Chinese international students who are currently studying abroad in diverse regions. Participants were mainland Chinese students who were currently pursuing varying degrees (e.g., bachelor, masters, Ph.D., postdoc) in differing cultural regions (e.g., United States, United Kingdom, Germany, and Australia, etc.). Our experiment was divided into three parts. The first part was cultural priming procedure. Based on previous work (Maddux and Galinsky, 2009), we adapted the multicultural experiences priming paradigm. Participants were randomly assigned into the following four conditions: studying abroad, traveling abroad, hometown, and control condition. Then participants were asked to write no less than 150 words in 5 min to describe their corresponding cultural experiences (see Supplementary Appendix A for instructions and materials). The second part was the measurement of tolerance for contradiction. Based on the initial measures of dialectical thinking (Peng and Nisbett, 1999), we collected scientific discoveries in the fields of natural sciences, social sciences and humanities in the scientific literature within the last 3 years, and then modified these scientific discoveries into two conflicting scientific statements (see Supplementary Appendix B for all the materials). If participants agree with both scientific statements in two opposing directions which are seemingly contradictory, that means participants deem both viewpoints are plausible and convincing. Lower scores on the differences between the endorsements of the two opposing statements means greater tolerance for contradiction. In the third part, we collected demographic variables. Participants were asked to indicate their gender, age, education level, socioeconomic status, current study abroad country and so forth.

## Results

**FIGURE 1:**
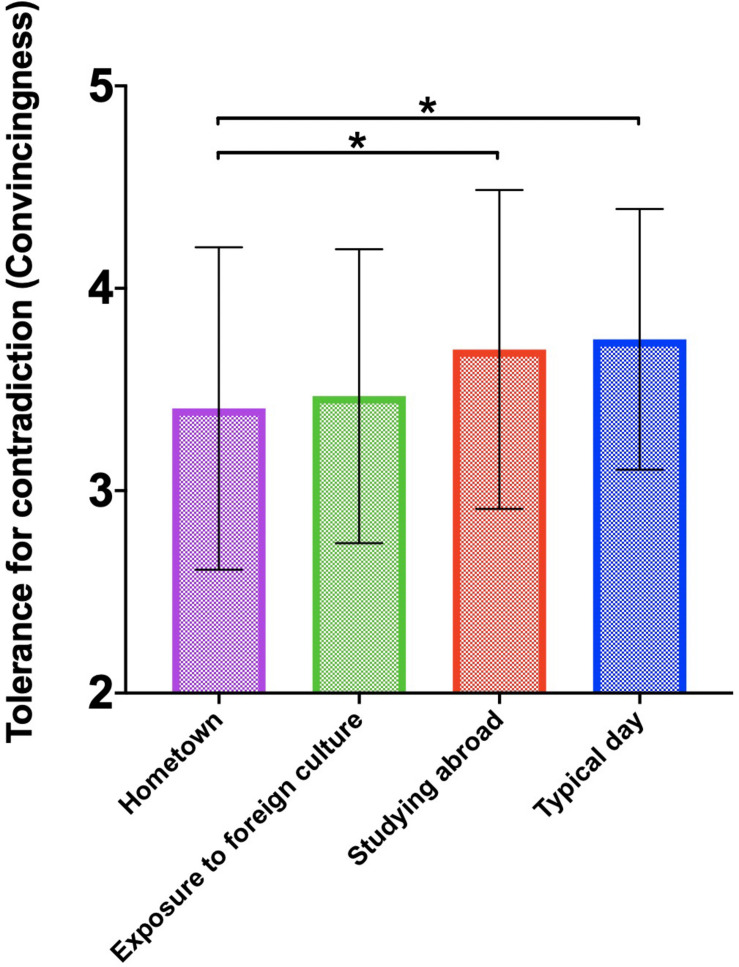
Tolerance for contradiction as measured by the convincingness of both statements among four priming conditions (^∗^*p* < 0.05).

**FIGURE 2:**
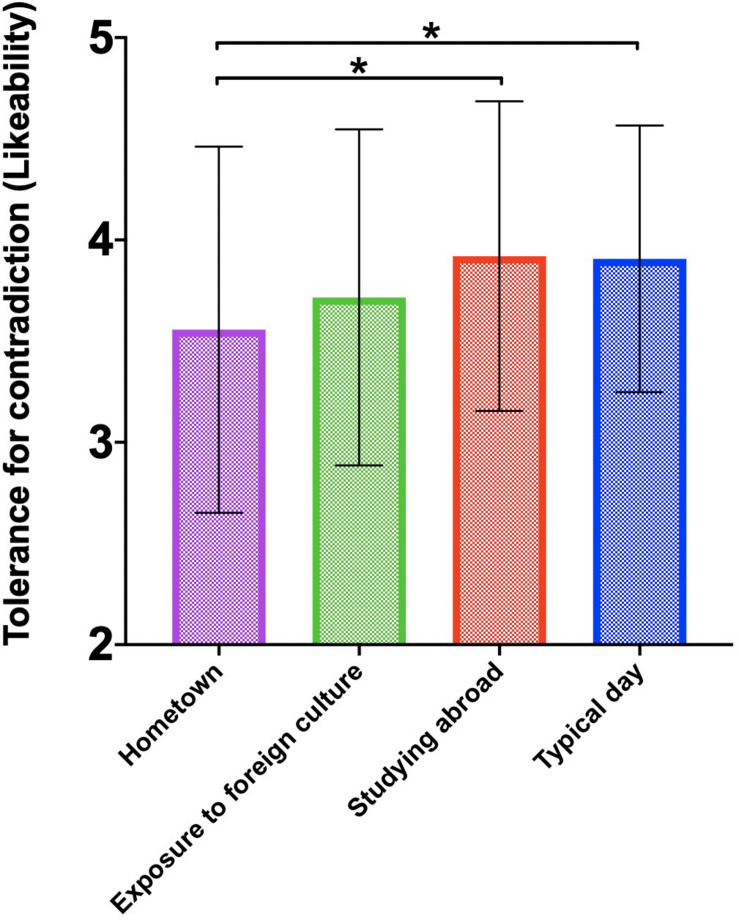
Tolerance for contradiction as measured by the like ability of both statements among four priming conditions (^∗^*p* < 0.05).

A total of 265 Chinese international students participated in our experiment. After deleting those who failed the attention check items (e.g., please choose 5 for this questions), 258 participants were included in our final analyses. According to Peng and Nisbett (1999), two scores represent the degree of tolerance for contradiction: convincingness (*M* = 3.55, SD = 0.76, Range = 0–6) and likeability (*M* = 3.75, SD = 0.83, Range = 0–6). We then conducted an ANOVA analysis. The results suggested that participants were more tolerant of contradictory scientific statements when primed with studying abroad experiences or typical day relative to hometown culture. In other words, when participants were manipulated to mentally stimulate the host culture in which they are currently studying, their degree of tolerance for conflicting viewpoints increased, at least temporarily. More specifically, they reported that two seemingly conflicting statements could co-exist and did not contradict each other. In other words, they reported that the two seemingly conflicting scientific findings were indeed both convincing, *F*(3,254) = 2.95, *p* < 0.05 and likeable, *F*(3,254) = 3.01, *p* < 0.05 (For subgroup differences please see Figures 1, 2). More specifically, for convincingness, the difference between studying abroad and hometown culture (*t* = 2.38, *p* < 0.05), as well as the difference between typical day and hometown culture (*t* = 2.21, *p* < 0.05) was significant. For likeability, the difference between studying abroad and hometown culture (*t* = 2.75, *p* < 0.05), as well as the difference between typical day and hometown culture (*t* = 2.10, *p* < 0.05) was significant. It is worth noting that the differences between priming studying abroad and priming typical day did not yield any meaningful results which was unexpected from our original theoretical formulations. We speculate that it is highly likely that priming typical day for the group of Chinese international students while they are studying abroad is naturally similar to priming their everyday life during studying abroad, which makes this control condition serve an equivalent function of priming studying abroad. Therefore, we deem both conditions as our experimental conditions and treat typical day priming as analogs as studying abroad priming. We did find that relative to hometown culture, participants were more likely to display dialectical thinking when primed with studying abroad or typical day.

## Discussion

Our current work is among the first to provide direct evidence to partially support the claim that Chinese internationals students’ studying abroad experience promotes dialectal thinking. Our data suggests that Chinese international students are more likely to accept contradictory scientific statements when primed with studying abroad experiences (comparing to priming with hometown culture). We have also observed consistent evidence between the two indicators of tolerance for contradiction: convincingness and likeability, meaning that Chinese international students are more likely to believe that the two seemingly conflicting scientific statements are both persuasive and rated them more favorably.

Why would priming studying abroad experiences enhance tolerance for contradiction? Previous work indicates that priming the yin-yang symbol among European Americans lead them to anticipate more changes in judgments and behaviors (Alter and Kwan, 2009). This finding suggests that temporarily priming salient cultural elements could shift participants’ dialectical thinking. Our results also indicate that when participants recall their studying abroad experiences in their respective host countries (e.g., United States, United Kingdom, Germany, and Australia, etc.), they are more likely to show greater tolerance of contradiction. We provide two possible interpretations for this finding. Why would experimentally inducing intensive foreign cultural experiences (i.e., studying abroad) rather than domestic cultural experiences (i.e., hometown) temporarily alter international students’ system of thought? The underlying processes may be partly explained by integrative complexity (Tadmor et al., 2012). Integrative complexity is a particular information-processing style which represents the ability to integrate differing perspectives or establish the conceptual connections among differentiated dimensions (Suedfeld and Bluck, 1993). With richer and deeper foreign experiences, especially in largely distinct cultural contexts, participants would be more likely to encounter multiple perspectives (e.g., Western vs. Eastern cultural traditions), incorporate complex or even competing systems of thought (e.g., holistic vs. analytical thinking), and differing value systems (e.g., individual rights and collective duties). They therefore have to reconcile contradictory viewpoints or even competing thought patterns. These demanding processes may play a role in promoting Chinese international students’ greater tolerance of contradiction, which manifested in endorsing competing scientific statements across a variety of domains (i.e., natural sciences, humanities and social sciences). As Chinese international students experience cultural shock, cultural conflict, and cultural integration through the course of cultural adaptation, their cognitive complexity may increase as they become more adept at reconciling competing perspectives and cultural differences. Another possible explanation might be that recalling studying abroad experiences pose cultural identity threats (Morris et al., 2011) which may induce reinforcements of previously existing Chinese way of thinking. Therefore, enhancement of dialectical thinking is a psychological outcome of cultural identity protection. These competing theoretical accounts await future work to validate and explore.

### Contributions and Implications

Our work has important contributions to the extant literature. First, past work has not looked at whether multicultural experiences could affect individuals’ ways of thinking. Our findings are among the first to demonstrate that studying abroad experiences does have an impact on Chinese international students’ dialectical thinking, specifically tolerance for contradiction. Second, our work adds to the growing literature that psychological outcomes of Chinese international students’ studying abroad experiences is not limited to academic achievements and mental health domains. Studying abroad experiences may elicit profound changes in International students’ cognitive, emotional, motivational and behavioral processes. Third, although cultural differences in dialectical thinking have been well-documented, dialectical thinking is a dynamic, flexible, and malleable construct. Dialectal thinking can therefore be shifted around even by subtle experimental manipulations. Our work has shed new light on the intersection among multicultural experiences, International students, and dialectical thinking. As we live in the era of globalization, more work is clearly needed to probe the important research question: how and why do multicultural experiences reshape individuals’ way of thinking, feeling, and behaving.

### Limitations and Future Directions

Some limitations also apply to our work. First, due to the difficulties of collecting data during the COVID-19 outbreak, our limited sample size reduced our statistical power. Future replication efforts should use a larger sample size to detect those effects. Second, as we only examined one of three indicators of dialectal thinking, future work should determine whether Chinese internationals students’ expectation of change and perception of interconnectedness can also be altered by priming studying abroad experiences. Third, since human behavior is the product of complex interactions between personal dispositions and contextual factors, future work should examine whether Chinese international students’ personality characteristics or cultural identity play a role in moderating the effects of multicultural experiences on their mental processes and behavioral patterns.

## Conclusion

Drawing upon a cultural priming experiment, we observed that Chinese international students were more likely to show tolerance for contradiction when they were primed with their host culture rather than primed with their home culture. Future work should examine the psychological underpinnings of this effect, potential mediators and boundary conditions, which we hope will encourage researchers, educators, and policy makers to facilitate both basic research and applied work in the domain of international education.

## Data Availability Statement

The raw data supporting the conclusions of this article will be made available by the authors, without undue reservation.

## Ethics Statement

The studies involving human participants were reviewed and approved by the Department of Psychology, Tsinghua University. The patients/participants provided their online versions of informed consent to participate in this study.

## Author Contributions

XH designed and carried out the study, collected and analyzed the data, and wrote the manuscript. YW and SL assisted with data collection. KP refined the research idea, supervised the study, and revised the manuscript. All authors contributed to the article and approved the submitted version.

## Conflict of Interest

The authors declare that the research was conducted in the absence of any commercial or financial relationships that could be construed as a potential conflict of interest.

## References

[B1] Alter A. L. Kwan V. S. Y. (2009). Cultural sharing in a global village: evidence for extracultural cognition in European Americans. *J. Pers. Soc. Psychol.* 96 742–760. 10.1037/a0014036 19309199

[B2] Billedo C. J. Kerkhof P. Finkenauer C. (2020). More facebook, less homesick? Investigating the short-term and long-term reciprocal relations of interactions, homesickness, and adjustment among international students. *Int. J. Intercult. Relat.* 75 118–131. 10.1016/j.ijintrel.2020.01.004

[B3] Buchan N. R. Grimalda G. Wilson R. Brewer M. Fatas E. Foddy M. (2009). Globalization and human cooperation. *Proc. Natl. Acad. Sci.U.S.A.* 106 4138–4142. 10.1073/pnas.0809522106 19255433 PMC2657440

[B4] Cao C. Meng Q. Shang L. (2018). How can Chinese international students’ host-national contact contribute to social connectedness, social support and reduced prejudice in the mainstream society? Testing a moderated mediation model. *Int. J. Intercult. Relat.* 63 43–52. 10.1016/j.ijintrel.2017.12.002

[B5] Cao J. Galinsky A. D. Maddux W. W. (2014). Does travel broaden the mind? Breadth of foreign experiences increases generalized trust. *Soc. Psychol. Pers. Sci.* 5 517–525. 10.1177/1948550613514456

[B6] Choy Y. Alon Z. (2018). The comprehensive mental health treatment of chinese international students: a case report. *J. Coll. Student Psychother.* 33 1–20. 10.1080/87568225.2018.1427513

[B7] Cubillos J. H. Ilvento T. (2012). The impact of study abroad on students’ self−efficacy perceptions. *Foreign Lang. Ann.* 45 494–511. 10.1111/j.1944-9720.2013.12002.x

[B8] Gieser J. D. (2015). A sociocultural investigation of identity: how students navigate the study abroad experience. *J. Coll. Student Dev.* 56 637–643. 10.1353/csd.2015.0060

[B9] Hazen H. D. Alberts H. C. (2006). Visitors or immigrants? international students in the United States. *Population Space Place* 12 201–216. 10.1002/psp.409

[B10] Hendrickson B. Rosen D. Aune R. K. (2011). An analysis of friendship networks, social connectedness, homesickness, and satisfaction levels of international students. *Int. J. Intercult. Relat.* 35 281–295. 10.1016/j.ijintrel.2010.08.001

[B11] Hong Y. Y. Morris M. W. Chiu C. Y. Benet-Martinez V. (2000). Multicultural minds: a dynamic constructivist approach to culture and cognition. *Am. Psychol.* 55:709. 10.1037/0003-066x.55.7.709 10916861

[B12] Hu X. Han Y. Yu F. Peng K. (2021). The double-edged sword effect of multicultural experiences: psychological consequences and boundary conditions. *Appl. Psychol.* 27, 1–10.

[B13] Huntington S. P. (1997). *The Clash of Civilizations and the Remaking of World Order.* New York, NY: Penguin Books India.

[B14] Kashima E. S. Loh E. (2006). International students acculturation: Effects of international, conational, and local ties and need for closure. *Int. J. Intercult. Relat.* 30 471–485. 10.1016/j.ijintrel.2005.12.003

[B15] Leung A. K.-y Maddux W. W. Galinsky A. D. Chiu C.-y (2008). Multicultural experience enhances creativity: the when and how. *Am. Psychol.* 63:169. 10.1037/0003-066X.63.3.169 18377107

[B16] Li G. Chen W. Duanmu J. L. (2010). Determinants of international students’ academic performance: a comparison between Chinese and other international students. *J. Stud. Int. Educ.* 14 389–405. 10.1177/1028315309331490

[B17] Liu M. (2009). Addressing the mental health problems of Chinese international college students in the United States. *Adv. Soc. Work* 10:69. 10.18060/164

[B18] Lu J. G. Quoidbach J. Gino F. Chakroff A. Maddux W. W. Galinsky A. D. (2017). The dark side of going abroad: how broad foreign experiences increase immoral behavior. *J. Pers. Soc. Psychol.* 112:1–16. 10.1037/pspa0000068 28032773

[B19] Maddux D. W. W. M. Lu D. J. G. Affinito M. S. J. Galinsky P. A. D. (2020). Multicultural experiences: a systematic review and new theoretical framework. *Acad. Manag. Ann.* 10.5465/annals.2019.0138

[B20] Maddux W. W. Galinsky A. D. (2009). Cultural borders and mental barriers: the relationship between living abroad and creativity. *J. Pers. Soc. Psychol.* 96:1047. 10.1037/a0014861 19379035

[B21] Maeder-Qian J. (2018). Intercultural experiences and cultural identity reconstruction of multilingual Chinese international students in Germany. *J. Multiling. Multicult. Dev.* 39 576–589. 10.1080/01434632.2017.1410161

[B22] Ma-Kellams C. Spencer-Rodgers J. Peng K. (2011). I am against us? Unpacking cultural differences in ingroup favoritism via dialecticism. *Pers. Soc. Psychol. Bull.* 37 15–27. 10.1177/0146167210388193 21084525

[B23] María Cubillo J. Sánchez J. Cerviño J. (2006). International students’ decision−making process. *Int. J. Educ. Manag.* 20 101–115. 10.1108/09513540610646091

[B24] Milstein T. (2005). Transformation abroad: sojourning and the perceived enhancement of self-efficacy. *Int. J. Intercult. Relat.* 29 217–238.

[B25] Mori S. C. (2011). Addressing the mental health concerns of international students. *J. Couns. Dev.* 78 137–144. 10.1002/j.1556-6676.2000.tb02571.x

[B26] Morris M. W. Mok A. Mor S. (2011). Cultural identity threat: the role of cultural identifications in moderating closure responses to foreign cultural inflow. *J. Soc. Issues* 67 760–773. 10.1111/j.1540-4560.2011.01726.x

[B27] Nisbett R. E. Peng K. Choi I. Norenzayan A. (2001). Culture and systems of thought: holistic versus analytic cognition. *Psychol. Rev.* 108:291. 10.1037/0033-295X.108.2.291 11381831

[B28] Oudenhoven J. P. V. Zee K. I. V. D. (2002). Predicting multicultural effectiveness of international students: the Multicultural Personality Questionnaire. *Int. J. Intercult. Relat.* 26 679–694. 10.1016/S0147-1767(02)00041-X

[B29] Peng K. Nisbett R. E. (1999). Culture, dialectics, and reasoning about contradiction. *Am. Psychol.* 54 741–754. 10.1037/0003-066X.54.9.741

[B30] Poyrazli S. Lopez M. D. (2007). an exploratory study of perceived discrimination and homesickness: a comparison of international students and American students. *J. Psychol.* 141 263–280. 10.3200/JRLP.141.3.263-280 17564257

[B31] Ritter S. M. Damian R. I. Simonton D. K. van Baaren R. B. Strick M. Derks J. (2012). Diversifying experiences enhance cognitive flexibility. *J. Exp. Soc. Psychol.* 48 961–964. 10.1016/j.jesp.2012.02.009

[B32] Ruble R. A. Zhang Y. B. (2013). Stereotypes of Chinese international students held by Americans. *Int. J. Intercult. Relat.* 37 202–211. 10.1016/j.ijintrel.2012.12.004

[B33] Sam D. L. (2001). Satisfaction with life among international students: an exploratory study. *Soc. Indic. Res.* 53 315–337. 10.1023/A:1007108614571

[B34] Sawir E. Marginson S. Deumert A. Nyland C. Ramia G. (2007). loneliness and international students: an Australian study. *J. Stud. Int. Educ.* 12 148–180. 10.1177/1028315307299699

[B35] Smith R. A. Khawaja N. G. (2011). A review of the acculturation experiences of international students. *Int. J. Intercult. Relat.* 35 699–713. 10.1016/j.ijintrel.2011.08.004

[B36] Sparkman D. J. Eidelman S. Blanchar J. C. (2016). Multicultural experiences reduce prejudice through personality shifts in openness to experience. *Eur. J. Soc. Psychol.* 46 840–853. 10.1002/ejsp.2189

[B37] Spencer-Oatey H. Xiong Z. (2006). Chinese students’ psychological and sociocultural adjustments to Britain: an empirical study. *Lang. Cult. Curriculum* 19 37–53. 10.1080/07908310608668753

[B38] Spencer-Rodgers J. Anderson E. Ma-Kellams C. Wang C. Peng K. (2018). “What is dialectical thinking? Conceptualization and measurement,” in *The Psychological and Cultural Foundations of East Asian Cognition: Contradiction, Change, and Holism*, eds Spencer-Rodgers J. Peng K. (Oxford: Oxford University Press), 1–34.

[B39] Suedfeld P. Bluck S. (1993). Changes in integrative complexity accompanying significant life events: historical evidence. *J. Pers. Soc. Psychol.* 64 124–130. 10.1037/0022-3514.64.1.124

[B40] Sümer S. Poyrazli S. Grahame K. (2014). Predictors of depression and anxiety among international students. *J. Couns. Dev.* 86 429–437. 10.1002/j.1556-6678.2008.tb00531.x

[B41] Tadmor C. T. Galinsky A. D. Maddux W. W. (2012). Getting the most out of living abroad: biculturalism and integrative complexity as key drivers of creative and professional success. *J. Pers. Soc. Psychol.* 103 520–542. 10.1037/a0029360 22823287

[B42] IIE. (2020). *The Annual Report of the Institution of International Education. The Power of International Education*.

[B43] Trice A. G. (2004). Mixing it up: international graduate students’ social interactions with American students. *J. Coll. Student Dev.* 45 671–687. 10.1353/csd.2004.0074

[B44] Wang C.-C. D. C. Mallinckrodt B. (2006). Acculturation, attachment, and psychosocial adjustment of Chinese/Taiwanese international students. *J. Couns. Psychol.* 53 422–433. 10.1037/0022-0167.53.4.422

[B45] Wang F. Peng K. Bai Y. Li R. Zhu Y. Sun P. (2016). The dorsal anterior cingulate cortex modulates dialectical self-thinking. *Front. Psychol.* 7:152. 10.3389/fpsyg.2016.00152 26903940 PMC4749714

[B46] Wei M. Heppner P. P. Mallen M. J. Ku T.-Y. Liao K. Y.-H. Wu T.-F. (2007). Acculturative stress, perfectionism, years in the United States, and depression among Chinese international students. *J. Couns. Psychol.* 54 385–394. 10.1037/0022-0167.54.4.385

[B47] Wong K. K. (2004). Are the learning styles of Asian international students culturally or contextually based? *Int. Educ. J.* 4 154–166.

[B48] Xu H. O’Brien W. H. Chen Y. (2019). Chinese international student stress and coping: a pilot study of acceptance and commitment therapy. *J. Contex. Behav. Sci.* 15 135–141. 10.1016/j.jcbs.2019.12.010

[B49] Yan K. Berliner D. C. (2011). Chinese international students in the United States: demographic trends, motivations, acculturation features and adjustment challenges. *Asia Pac. Educ. Rev.* 12 173–184. 10.1007/s12564-010-9117-x

[B50] Zhuang X. Y. Wong D. F. K. Ng T. K. Poon A. (2019). Effectiveness of mental health first aid for Chinese-speaking international students in Melbourne. *Res. Soc. Work Pract.* 30:104973151989039. 10.1177/1049731519890398

[B51] Zimmermann J. Neyer F. J. (2013). Do we become a different person when hitting the road? Personality development of sojourners. *J. Pers. Soc. Psychol.* 105 515–530. 10.1037/a0033019 23773042

