# Supplementary material for: Do Experiences Studying Abroad Promote Dialectical Thinking? Empirical Evidence From Chinese International Students
Source: Front Psychol. 2021 May 28;12:595935. doi: 10.3389/fpsyg.2021.595935 (PMC8195590; doi:10.3389/fpsyg.2021.595935)
Supplement: Supplementary file 1 [file Dat_Sheet_1.DOCX]

Appendix A:

**Instructions for the Cultural Priming Procedure**

**（Mandarin version and English version）**

1a: 留学经历

你目前正在哪个国家留学？当你在该国留学的时候，你在多大程度上沉浸在该国的文化中？请描述一下你在该国的文化经历。（例如，你会吃什么食物？去过哪些地方？如何度过闲暇时光？参加了什么课程？是否在学习该国语言？读过哪些和该国文化相关的书籍，和当地人的社交经历？参加了哪些文化活动等）（以上举例仅供参考，请大家结合实际生活经历描述）请在5分钟内写下不少于150字。

1b: Studying abroad

Which country are you currently studying in? When you study abroad in that country, how much are you immersed in the culture of that country? Please describe your cultural experiences in that country. (For example, what food do you eat? Where do you go? How do you spend your free time? What courses have you taken? Are you learning the language of the country? What books have you read about the country's culture, and the social experiences of the locals? What cultural activities have you participated in? (The above examples are for reference only, please describe them based on your actual life experiences) Please write no less than 150 words within 5 minutes.

2a:旅行经历

你曾经去过哪些国家旅游或生活？其中文化体验最深刻的是哪一个国家？你在多大程度上接触了该国的文化？请描述一下你在该国的文化体验。（例如，你会吃什么食物？去过哪些地方？如何度过闲暇时光？是否在学习该国语言？读过哪些和该国文化相关的书籍，和当地人的社交经历？参加了哪些文化活动等）（以上举例仅供参考，请大家结合实际生活经历描述）请在5分钟内写下不少于150字。

2b: Travelling abroad

Which countries have you traveled to or lived in? In which country did you have most profound cultural experience? To what extent have you been exposed to the country’s culture? Please describe your cultural experience in that country. (For example, what food did you eat? Where did you go? How did you spend your free time? Were you learning the language of the country? What books have you read about the country’s culture, and the social experiences of the locals? What cultural activities did you participate in? etc.) (The above examples are for reference only, please describe based on your actual life experiences) Please write no less than 150 words within 5 minutes.

3a: 家乡文化

你的家乡是哪个城市/区县，你的家乡文化是怎样的？请描述一下你的家乡文化特色。（例如，你在家乡会吃什么食物？去过家乡哪些文化景点？和家乡当地人的社交经历？参加过哪些的家乡的文化活动等）请在5分钟内写下不少于150字。

3b: Hometown Culture

Which city/district is your hometown and what is your hometown culture? Please describe the cultural characteristics of your hometown. (For example, what kind of food do you eat in your hometown? What cultural attractions have you visited in your hometown? Social experiences with locals in your hometown? What cultural activities in your hometown have you participated in, etc.) Please write no less than 150 words within 5 minutes.

4a: 典型一天

请描述你典型的一天是如何度过的。（例如某天你做了哪些事情、你的感受如何）。请在5分钟内写下不少于150字。）

4b: Typical Day

Please describe how you spend a typical day. (For example, what did you do on a certain day, how did you feel). Please write no less than 150 words within 5 minutes. )

Appendix B:

Scientific Statements of Contradictory Findings

（Mandarin version and English version）

1a:

结论A：研究发现大脑越大的人越聪明。

结论B：研究发现人的聪明程度与大脑体积无关。

1b:

Conclusion A: Research has found that people with bigger brains are smarter.

Conclusion B: Research has found that people’s intelligence has nothing to do with brain size.

2a:

结论A：研究表明，儿童使用手机电视电脑等越频繁，就越容易患多动症。

结论B：研究表明，儿童经常使用手机电视电脑等媒体可以增加他们的专注度。

2b:

Conclusion A: Research shows that the more frequently children use mobile phones, televisions, computers, etc., the more likely they are to develop ADHD.

Conclusion B: Research shows that children's frequent use of media such as mobile phones, televisions and computers can increase their ability of concentration.

3a:

结论A：研究表明，全球变暖会加剧经济的不平衡。

结论B：研究表明，全球变暖会使世界经济趋向平衡。

3b:

Conclusion A: Research shows that global warming will exacerbate economic imbalances.

Conclusion B: Research shows that global warming will make the world economy more balanced.

4a:

结论A：研究表明，向消费者提供有关商品使用发生故障概率的信息会显著降低他们支付额外保修费用的意愿。
结论B：研究表明，向消费者提供有关商品使用发生故障概率的信息会显著提升他们支付额外保修费用的意愿。

4b:

Conclusion A: Studies have shown that providing consumers with information about the probability of failure in product use will significantly reduce their willingness to pay for additional warranty costs.

Conclusion B: Studies have shown that providing consumers with information about the probability of failure in product use will significantly increase their willingness to pay for additional warranty costs.

5a:

结论A：研究表明，女性平均工资低于男性。
结论B：研究表明，男性平均工资低于女性。

5b:

Conclusion A: Research shows that women’s average wages are lower than men’s.

Conclusion B: Research shows that the average salary of men is lower than that of women.

6a:

结论A：研究表明，网约车增加了城市交通恶化的程度。
结论B：研究表明，网约车能改善城市交通拥堵情况。

6b:

Conclusion A: Research shows that online car-hailing increases the degree of urban traffic deterioration.

Conclusion B: Research shows that online car-hailing can improve urban traffic congestion.

7a:

结论A：研究表明，冰川融化导致水中二氧化碳释放，使大气中二氧化碳浓度升高。
结论B：研究表明，冰川融化，大气中水蒸气增加，使二氧化碳在大气中的浓度减少。

7b:

Conclusion A: Studies have shown that the melting of glaciers causes the release of carbon dioxide in the water, which increases the concentration of carbon dioxide in the atmosphere.

Conclusion B: Studies have shown that when glaciers melt, water vapor in the atmosphere increases, which reduces the concentration of carbon dioxide in the atmosphere.

8a:

结论A：研究表明，秸秆肥料在田间逐渐被分解，会产生大量温室气体。
结论B：研究表明，秸秆肥料被田间的植物吸收，促进植物光合作用吸收温室气体并产生氧气。

8b:

Conclusion A: Studies have shown that straw fertilizer is gradually decomposed in the field, which will produce a lot of greenhouse gases.

Conclusion B: Studies have shown that straw fertilizer is absorbed by plants in the field, which promotes photosynthesis of plants to absorb greenhouse gases and produce oxygen.

9a:

结论A：研究表明，水珠沿湿润的棉线向下滴落时，即使棉线上水流速度加快，水珠的尺寸也不会发生变化。
结论B：研究表明，水珠沿湿润的棉线向下滴落，随着棉线上水流速度的增加，水珠的尺寸会显著增大。

9b:

Conclusion A: Studies have shown that when water drops drop down along the wet cotton thread, the size of the water drops will not change even if the speed of the water on the cotton thread increases.

Conclusion B: Studies have shown that water drops drop down along the wet cotton thread. As the water flow speed on the cotton thread increases, the size of the water drops will increase significantly.

10a:

结论A：研究表明，中国PM2.5减少导致其他空气污染的增加。
结论B：研究表明，中国PM2.5减少使得空气质量显著变好。

10b:

Conclusion A: Studies have shown that the reduction of PM2.5 in China has led to an increase in other air pollution.

Conclusion B: Studies have shown that the reduction of PM2.5 in China has significantly improved air quality.

11a:

结论A：研究表明，岛屿上体型细长的蜥蜴由于体轻更容易被大风刮跑。
结论B：研究表明，为了抵御大风减少阻力，岛屿上蜥蜴不断进化为拥有细长体型的物种。

11b:

Conclusion A: Studies have shown that slender lizards on islands are more likely to be blown away by high winds due to their light weight.

Conclusion B: Studies have shown that in order to withstand high winds and reduce resistance, lizards on the islands continue to evolve into slender species.

12a:

结论A：研究表明，掠食性鱼类入侵某淡水湖时，会导致湖内原有鱼类减少，造成了鱼鹰缺少食物。
结论B：研究表明，掠食性鱼类入侵某淡水湖时，虽然使湖内原有鱼类减少，但并不会造成鱼鹰的食物稀缺。

12b:

Conclusion A: Studies have shown that when predatory fish invade a freshwater lake, it will reduce the number of original fish in the lake, causing the osprey to lack food.

Conclusion B: Studies have shown that when predatory fish invade a freshwater lake, although the original fish in the lake will be reduced, it will not cause food scarcity for the osprey.
